# Supplementary material for: Hepatic nutrient and hormone signaling to mTORC1 instructs the postnatal metabolic zonation of the liver
Source: Nat Commun. 2024 Mar 18;15:1878. doi: 10.1038/s41467-024-46032-1 (PMC10948770; doi:10.1038/s41467-024-46032-1)
Supplement: Supplementary file 1 — Supplementary Information [file 41467_2024_46032_MOESM1_ESM.pdf]

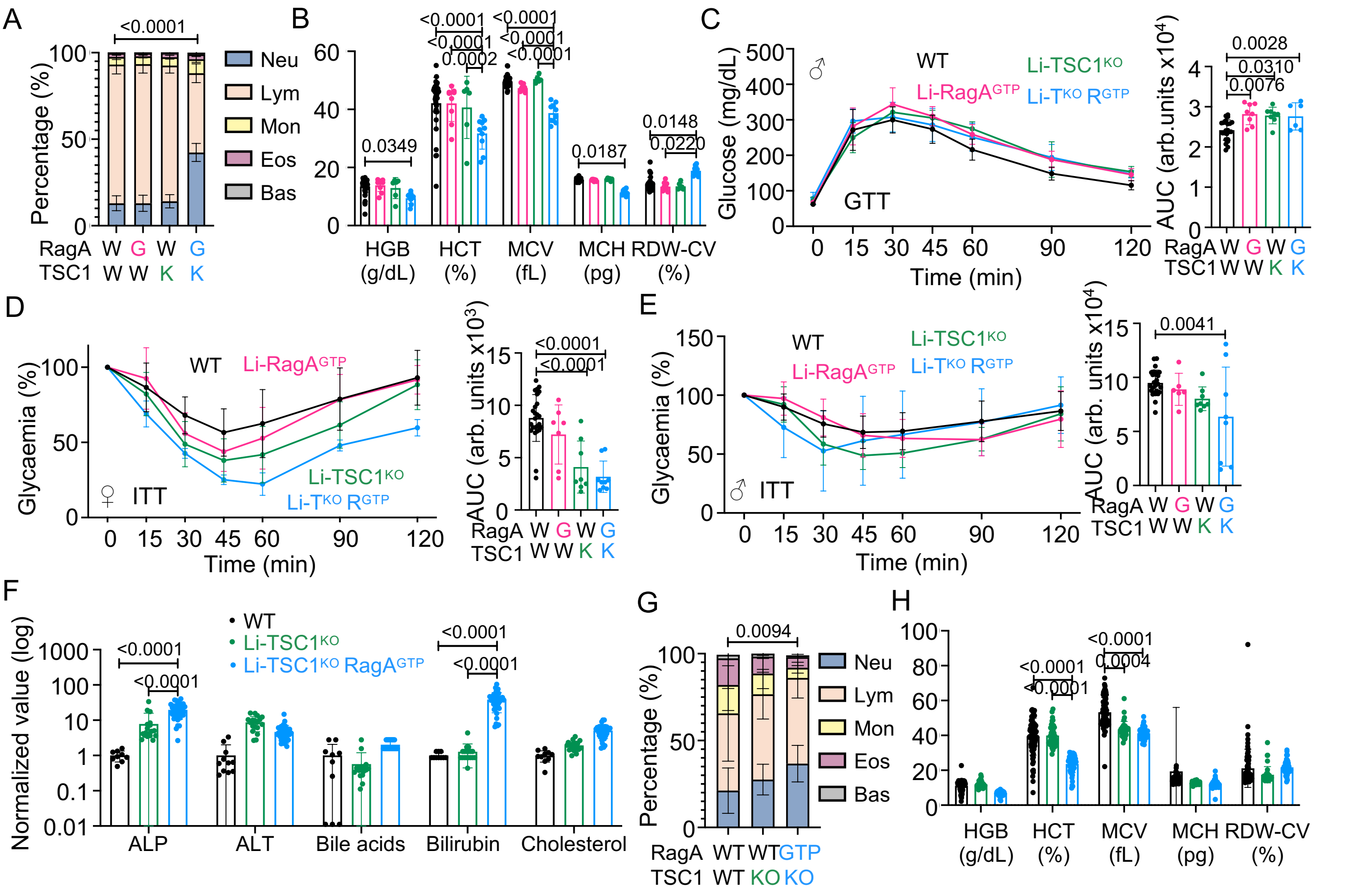

Supplementary Figure 1. Related to Figure 1. A. Percentage of neutrophils (Neu), lymphocytes (Lym), monocytes (Mon), eosinophils (Eos) and basophils (Bas) in adult wild-type (n=33), Li-RagA^{GTP} (n=9), Li-TSC1^{KO} (n=6) and Li-TSC1^{KO}RagA^{GTP} (n=10) mice. Statistical significance was calculated with chi-squared test. **B.** Levels of hemoglobin (HGB), hematocrit (HCT), mean corpuscular volume (MCV), mean corpuscular hemoglobin (MCH) and red cell distribution width (RDW-CV) in adult wild-type (n=33), Li-RagA^{GTP} (n=9), Li-TSC1^{KO} (n=6) and Li-TSC1^{KO}RagA^{GTP} (n=10) mice. Statistical significance was calculated by using 2way ANOVA with Tukey's multiple comparisons test. **C.** Glucose tolerance test (GTT) of 7- to 14-week-old wild-type (n=23), Li-RagA^{GTP} (n=8), Li-TSC1^{KO} (n=8) and Li-TSC1^{KO}RagA^{GTP} (n=6) males and area under the curve (AUC) of glucose tolerance test. Statistical significance was calculated by using 1way ANOVA with Tukey's multiple comparisons test. **D.** Insulin tolerance test (ITT) of 11- to 18-week-old wild-type (n=26), Li-RagA^{GTP} (n=7), Li-TSC1^{KO} (n=7) and Li-TSC1^{KO}RagA^{GTP} (n=6) females and area under the curve (AUC) of insulin tolerance test. Statistical significance was calculated by using 1way ANOVA with Tukey's multiple comparisons test. **E.** Insulin tolerance test (ITT) of 12- to 18-week-old wild-type (n=27), Li-RagA^{GTP} (n=6), Li-TSC1^{KO} (n=8) and Li-TSC1^{KO}RagA^{GTP} (n=8) female and area under the curve (AUC) of insulin tolerance test. Statistical significance was calculated by using 1way ANOVA with Tukey's multiple comparisons test. **F.** Levels of circulating alanine aminotransferase (ALT), alkaline phosphatase (ALP), bile acids, bilirubin and cholesterol were measured in aged *ad libitum* wild-type (n=11), Li-TSC1^{KO} (n=18) and Li-TSC1^{KO}RagA^{GTP} (n=38) mice. Values were made relative to the average level in wild-type mice and presented in log_{10} scale. Statistical significance was calculated by using 2way ANOVA with Tukey's multiple comparisons test. **G.** Percentage of neutrophils (Neu), lymphocytes (Lym), monocytes (Mon), eosinophils (Eos) and basophils (Bas) in aged wild-type (n=73), Li-TSC1^{KO} (n=37) and Li-TSC1^{KO}RagA^{GTP} (n=53) mice. Statistical significance was calculated with chi-squared test. **H.** Levels of hemoglobin (HGB), hematocrit (HCT), mean corpuscular volume (MCV), mean corpuscular hemoglobin (MCH) and red cell distribution width (RDW-CV) in aged wild-type (n=73), Li-TSC1^{KO} (n=37) and Li-TSC1^{KO}RagA^{GTP} (n=53) mice. Statistical significance was calculated by using 2way ANOVA with Tukey's multiple comparisons test.

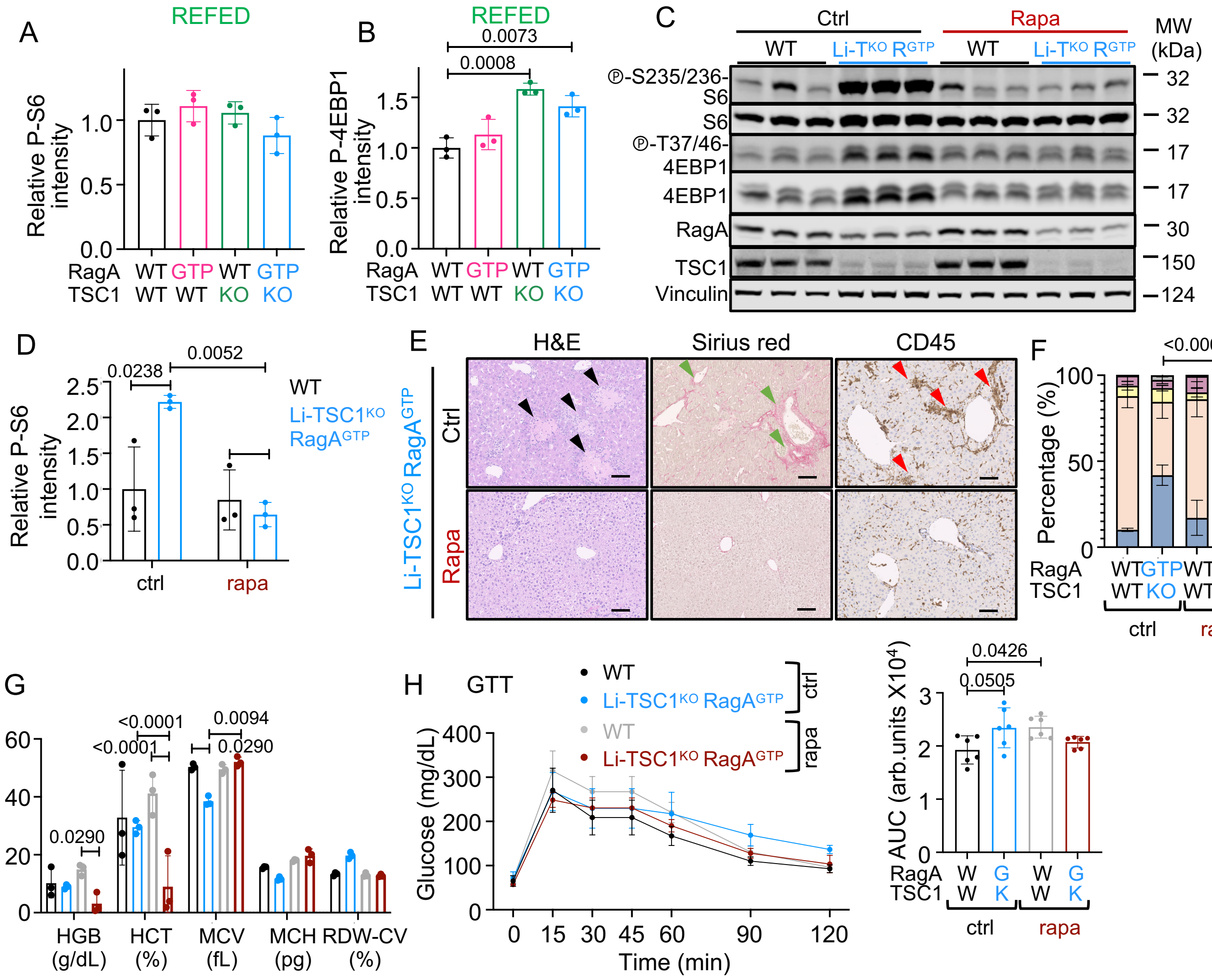

Supplementary Figure 2. Related to Figure 2. A. Levels of Phospho-S235/236-S6 from refed state on each lane from Fig 2a are relative to vinculin levels and presented normalized to the average level of refed wild-type mice. Statistical significance was calculated by using 1way ANOVA with Tukey's multiple comparisons test. **B.** Levels of Phospho-T37/46-4EBP1 from refed state on each lane from Fig 2a are relative to vinculin levels and presented normalized to the average level of refed wild-type mice. Statistical significance was calculated by using 1way ANOVA with Tukey's multiple comparisons test. **C.** 18- to 20-week-old control and rapamycin-treated wild-type and Li-TSC1^{KO}RagA^{GTP} male mice were sacrificed *ad libitum*. Protein lysates from the liver were immunoblotted for the indicated proteins. **D.** Levels of Phospho-S235/236-S6 on each lane from Supp Fig 2c are relative to vinculin levels and presented normalized to the average level of wild-type mice in control condition. Statistical significance was calculated by using 2way ANOVA with Sidak's multiple comparisons test. **E.** Representative hepatic hematoxylin and eosin (H&E), Sirius red staining and CD45 immunohistochemistry of Li-TSC1^{KO}RagA^{GTP} control and Li-TSC1^{KO}RagA^{GTP} rapamycin mice. Black, green and red arrowheads indicate necrotic, fibrotic and inflammatory areas, respectively. Scale bar 100 μm. **F.** Percentage of neutrophils (Neu), lymphocytes (Lym), monocytes (Mon), eosinophils (Eos) and basophils (Bas) in 18- to 20-week-old WT control (n=3), WT rapamycin (n=3), Li-TSC1^{KO}RagA^{GTP} control (n=3) and Li-TSC1^{KO}RagA^{GTP} rapamycin (n=3) mice. Statistical significance was calculated with chi-squared test. **G.** Levels of hemoglobin (HGB), hematocrit (HCT), mean corpuscular volume (MCV), mean corpuscular hemoglobin (MCH) and red cell distribution width (RDW-CV) in 18- to 20-week-old WT control (n=3), WT rapamycin (n=3), Li-TSC1^{KO}RagA^{GTP} control (n=3) and Li-TSC1^{KO}RagA^{GTP} rapamycin (n=3) mice. Statistical significance was calculated by using 2way ANOVA with Tukey's multiple comparisons test. **H.** Glucose tolerance test (GTT) of 9- to 12-week-old WT control (n=6), WT rapamycin (n=6), Li-TSC1^{KO}RagA^{GTP} control (n=6) and Li-TSC1^{KO}RagA^{GTP} rapamycin (n=6) mice and Area under the curve (AUC) of glucose tolerance test. Statistical significance was calculated by using 2way ANOVA with Sidak's multiple comparisons test.

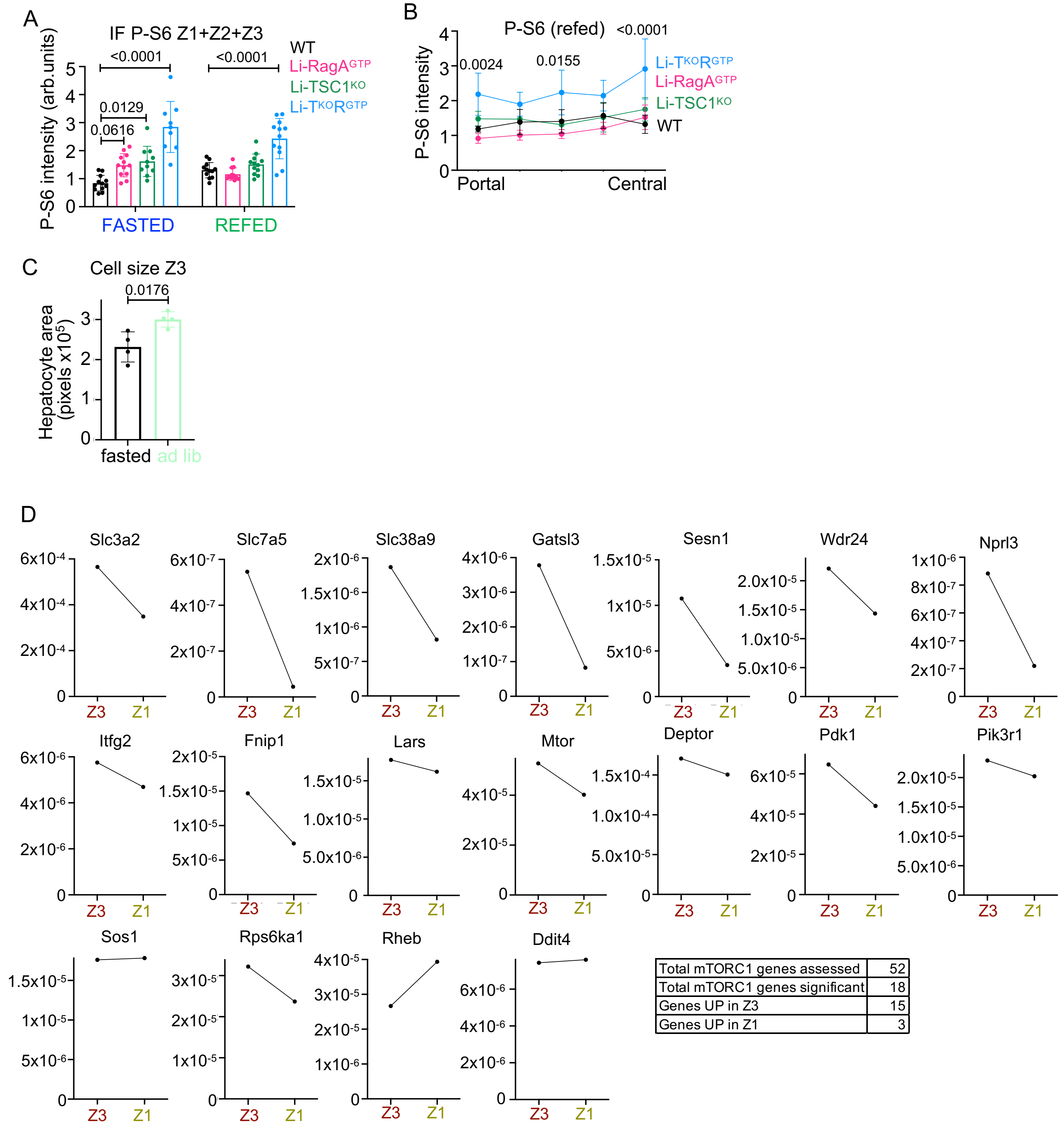

Total mTORC1 genes assessed	52
Total mTORC1 genes significant	18
Genes UP in Z3	15
Genes UP in Z1	3

Supplementary Figure 3. Related to Figure 3. A. Quantification of the Phospho-S240/244-S6 intensity in the hepatocytes of Z1, Z2 and Z3 of wild-type (n=4), Li-RagA^{GTP} (n=4), Li-TSC1^{KO} (n=4) and Li-TSC1^{KO}RagA^{GTP} (n=3) mice fasted during 24 hours or fasted during 24 hours followed by a 2-hours of refed. **B.** Quantification of the Phospho-S240/244-S6 intensity of wild-type (n=4), Li-RagA^{GTP} (n=4), Li-TSC1^{KO} (n=4) and Li-TSC1^{KO}RagA^{GTP} (n=3) mice fasted during 24 hours followed by 2 hours of refed from portal to central zones. Statistical significance was calculated by using 2way ANOVA with Tukey's multiple comparisons test. **C.** Quantification of hepatocyte area in the zone 3 of the liver of wild-type mice fasted during 24 hours (n=4) or fasted during 24 hours followed by a 2-hours of refed (n=4). Statistical significance was calculated by using unpaired two-tailed t-test. **D.** Expression gradient between hepatic Z3 and Z1 of components and regulators of the mTORC1 signaling pathway (meta analysis performed from Halpern et al 2017 data). Z3 was defined as the average from Layers 1,2 and 3 and Z1 was defined as the average from Layers 7, 8 and 9 from Halpern et al 2017. mTORC1 genes have been considered as significantly zonated based on the criteria defined by Halpern et al 2017 (q-value <0.2); 15/18 (Slc3a2, Slc7a5, Slc38a9, Gatsl3, Sesn1, Wdr24, Nprl3, Itfg2, Fnip1, Lars, Mtor, Deptor, Pdj1, Pik3r1,Rps6ka1) transcripts have a Z3-to-Z1 decrease in expression, and 3/18 (Rheb, Sos1 and Ddit4) with Z1-to-Z3 decrease.

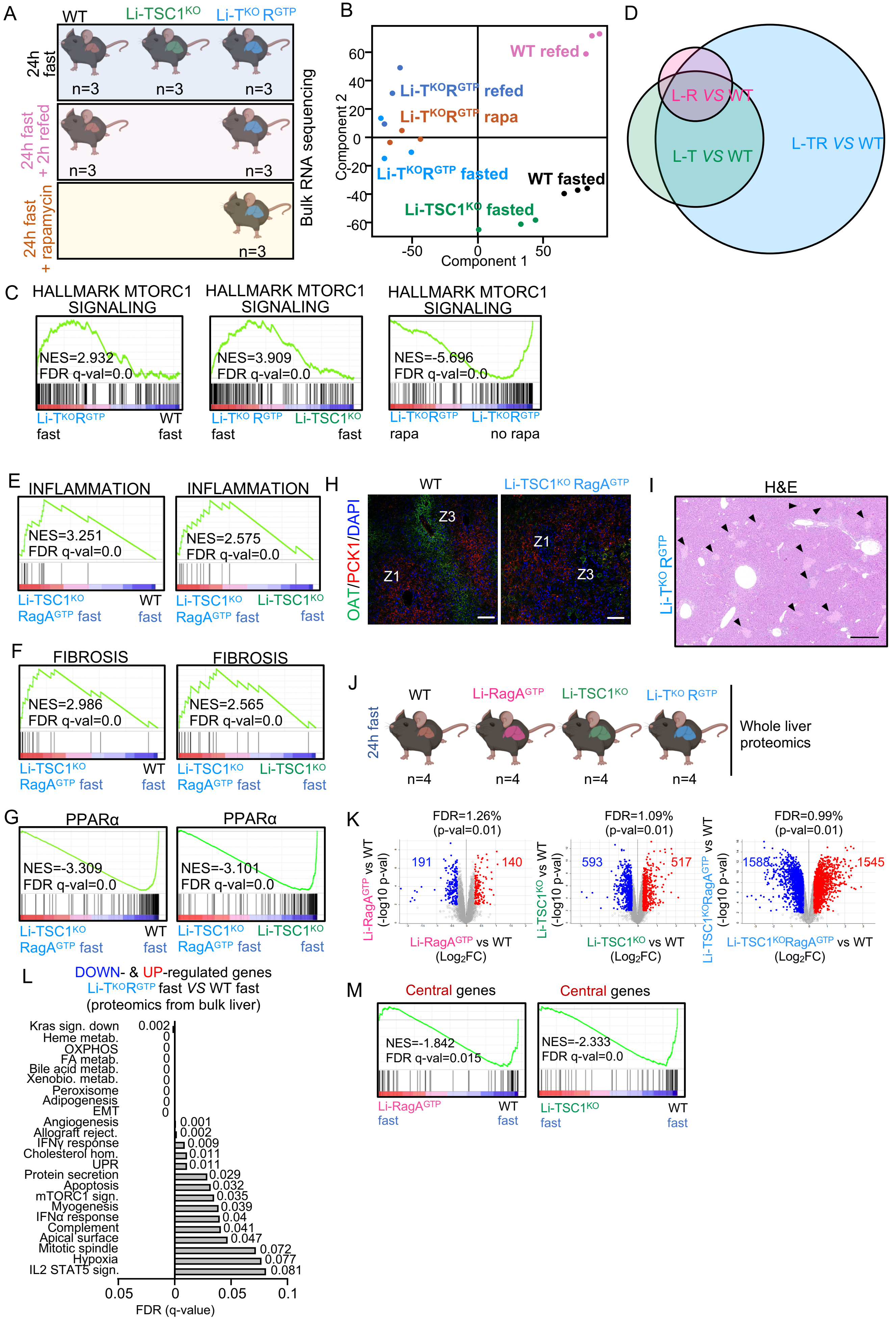

A
WT
Li-TSC1KO
Li-TKO RGTP
24h fast
n=3
24h fast + 2h refed
24h fast + rapamycin
Bulk RNA sequencing
B
Component 2
Component 1
WT refed
Li-TKORGTP refed
Li-TKORGTP rapa
Li-TKORGTP fasted
WT fasted
Li-TSC1KO fasted
D
L-R VS WT
L-T VS WT
L-TR VS WT
C
HALLMARK MTORC1 SIGNALING
NES=2.932
FDR q-val=0.0
Li-TKORGTP fast
WT fast
NES=3.909
FDR q-val=0.0
Li-TKO RGTP fast
Li-TSC1KO fast
NES=-5.696
FDR q-val=0.0
Li-TKORGTP rapa
Li-TKORGTP no rapa
E
INFLAMMATION
NES=3.251
FDR q-val=0.0
Li-TSC1KO RagAGTP fast
WT fast
NES=2.575
FDR q-val=0.0
Li-TSC1KO RagAGTP fast
Li-TSC1KO fast
H
WT
Li-TSC1KO RagAGTP
OAT/PCK1/DAPI
Z1
Z3
I
H&E
Li-TKO RGTP
F
FIBROSIS
NES=2.986
FDR q-val=0.0
NES=2.565
FDR q-val=0.0
J
24h fast
WT
Li-RagAGTP
Li-TSC1KO
Li-TKO RGTP
n=4
Whole liver proteomics
G
PPARα
NES=-3.309
FDR q-val=0.0
NES=-3.101
FDR q-val=0.0
K
FDR=1.26% (p-val=0.01)
191
140
Li-RagAGTP vs WT (-log10 p-val)
Li-RagAGTP vs WT (Log2FC)
FDR=1.09% (p-val=0.01)
593
517
Li-TSC1KO vs WT (-log10 p-val)
Li-TSC1KO vs WT (Log2FC)
FDR=0.99% (p-val=0.01)
1588
1545
Li-TSC1KORagAGTP vs WT (-log10 p-val)
Li-TSC1KORagAGTP vs WT (Log2FC)
L
DOWN- & UP-regulated genes
Li-TKORGTP fast VS WT fast
(proteomics from bulk liver)
Kras sign. down 0.002
Heme metab. 0
OXPHOS 0
FA metab. 0
Bile acid metab. 0
Xenobio. metab. 0
Peroxisome 0
Adipogenesis 0
EMT 0
Angiogenesis 0.001
Allograft reject. 0.002
IFNγ response 0.009
Cholesterol hom. 0.011
UPR 0.011
Protein secretion 0.029
Apoptosis 0.032
mTORC1 sign. 0.035
Myogenesis 0.039
IFNα response 0.04
Complement 0.041
Apical surface 0.047
Mitotic spindle 0.072
Hypoxia 0.077
IL2 STAT5 sign. 0.081
0.05
0
0.05
0.1
FDR (q-value)
M
Central genes
NES=-1.842
FDR q-val=0.015
Li-RagAGTP fast
WT fast
NES=-2.333
FDR q-val=0.0
Li-TSC1KO fast
WT fast

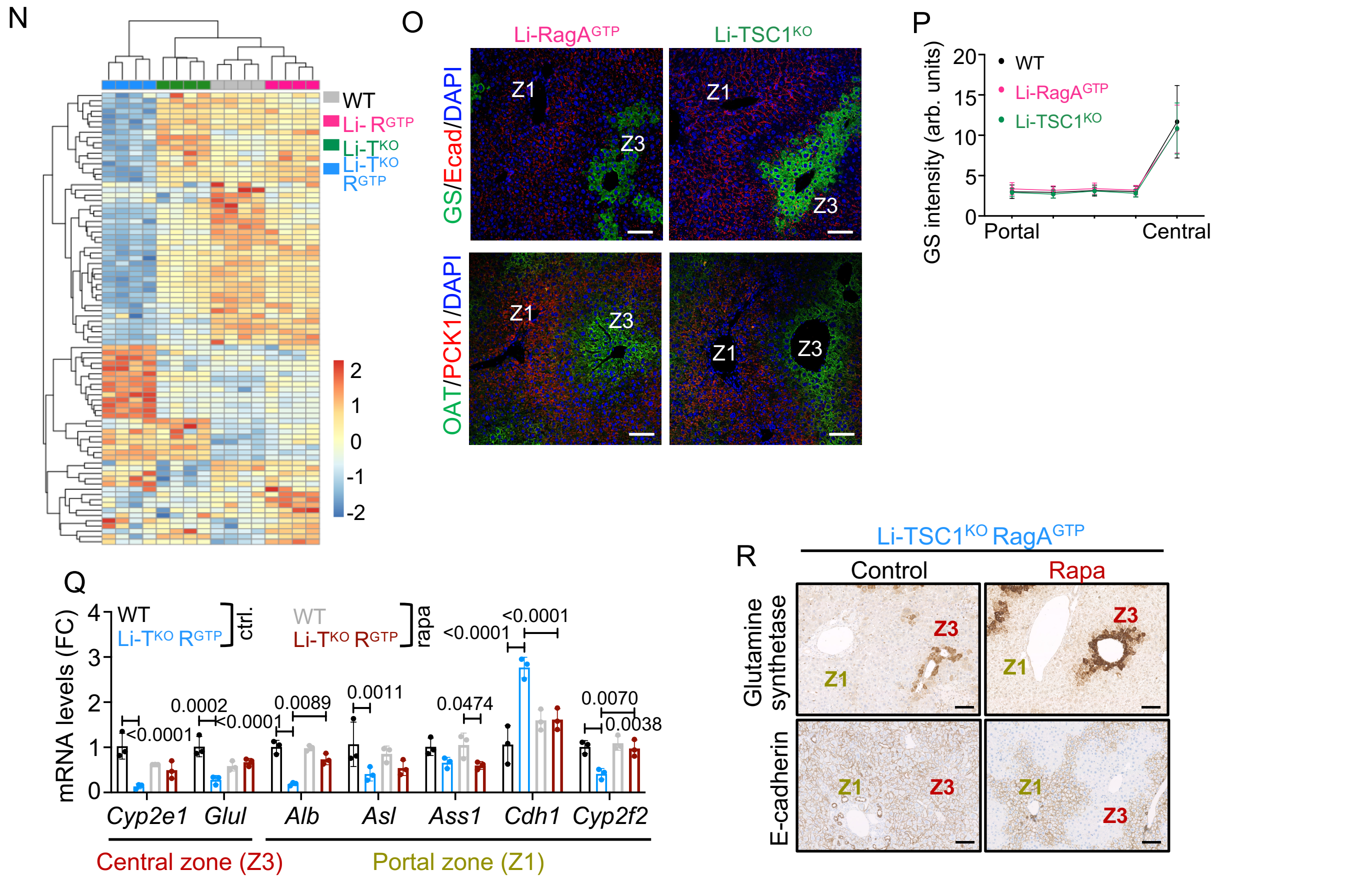

Supplementary Figure 4. Related to Figure 4. **A.** Schematic representation of the experimental setting for liver transcriptomics from 10- to-26-week-old mice. Number of samples in each condition is indicated for the three genotypes. Picture generated with BioRender. **B.** PCA of the transcriptomic profiles of the samples. Each dot represents individual biological replicates. **C.** Enrichment plots for the "mTORC1 signaling" gene set from Hallmark for the indicated comparisons. **D.** Proportional Venn diagram showing the overlap of significantly de-regulated proteins between the comparisons of Li-TSC1^{KO}RagA^{GTP} *VS* wild-type, Li-TSC1^{KO} *VS* wild-type and Li-RagA^{GTP} *VS* wild-type. **E.** Enrichment plots for the "Inflammation" gene set for the indicated comparisons. **F.** Enrichment plots for the "Fibrosis" gene set for the indicated comparisons. **G.** Enrichment plots for the "PPAR alpha" gene set for the indicated comparisons. NES: normalized enrichment score; FDR: false discovery rate. **H.** Representative pictures of immunofluorescence against Ornithine aminotransferase (Zone 3) together with Phosphoenolpyruvate Carboxykinase 1 (Zone 1) in the liver of adult wild-type and Li-TSC1^{KO} RagA^{GTP} mice. Zone 1 and Zone 3 are highlighted in each image. Scale bar 100 µm. **I.** Representative hepatic H&E staining of Li-TSC1^{KO}RagA^{GTP} mice. Black arrowheads indicate necrotic areas, which are not associated with Z1 or Z3. Scale bar 500 µm. **J.** Schematic representation of the experimental setting for liver proteomics from 16- to-26-week-old female mice. Number of samples in each condition is indicated for the four genotypes. Picture generated with BioRender. **K.** Volcano plots highlighting significantly different protein levels in livers from Li-RagA^{GTP} (n=4) *versus* wild-type (n=4) mice (left), Li-TSC1^{KO} (n=4) *versus* wild-type (n=4) mice (center) and Li-TSC1^{KO}RagA^{GTP} (n=4) *versus* wild-type (n=4) mice (right). **L.** Representation of the false discovery rates (FDR) from the top Hallmark gene sets enriched and depleted in livers from Li-TSC1^{KO}RagA^{GTP} (n=4) *versus* wild-type (n=4) mice. **M.** Enrichment of gene sets related to central signatures in proteomics from wild-type (n=4) and Li-RagA^{GTP} (n=4) livers (right) and from wild-type (n=4) and Li-TSC1^{KO} (n=4) livers (left). NES: normalized enrichment score; FDR: false discovery rate. **N.** Hierarchical clustering Heatmap diagram representing protein levels of Li-TSC1^{KO}RagA^{GTP} (n=4), Li-TSC1^{KO} (n=4), Li-RagA^{GTP} (n=4) and wild-type (n=4) livers normalized by z-score. **O.** Representative pictures of immunofluorescence against Glutamine synthetase (Zone 3) together with E-cadherin (Zone 1) or Ornithine aminotransferase (Zone 3) together with Phosphoenolpyruvate Carboxykinase 1 (Zone 1) in the liver of adult Li-RagA^{GTP} and Li-TSC1^{KO} mice. Zone 1 and Zone 3 are highlighted in each image. **P.** Quantification of the Glutamine synthetase intensity of wild-type (n=4), Li-RagA^{GTP} (n=4) and Li-TSC1^{KO} (n=4) livers from portal to central zone. Statistical significance was calculated by using 2way ANOVA with Tukey's multiple comparisons test. **Q.** RT-qPCR of livers from 18- to 20-week-old wild-type control (n=3), wild-type rapamycin (n=3), Li-TSC1^{KO}RagA^{GTP} control (n=3) and Li-TSC1^{KO}RagA^{GTP} rapamycin (n=3) male mice. Expression levels of the indicated genes involved in zonation relative to the average level in wild-type control mice. β-actin was used as housekeeping gene. Statistical significance was calculated by using 2way ANOVA with Tukey's multiple comparisons test. **R.** Representative pictures of immunohistochemistry against Glutamine synthetase or E-cadherin in the liver of control and rapamycin-treated Li-TSC1^{KO}RagA^{GTP} mice. Scale bar 100 µm.

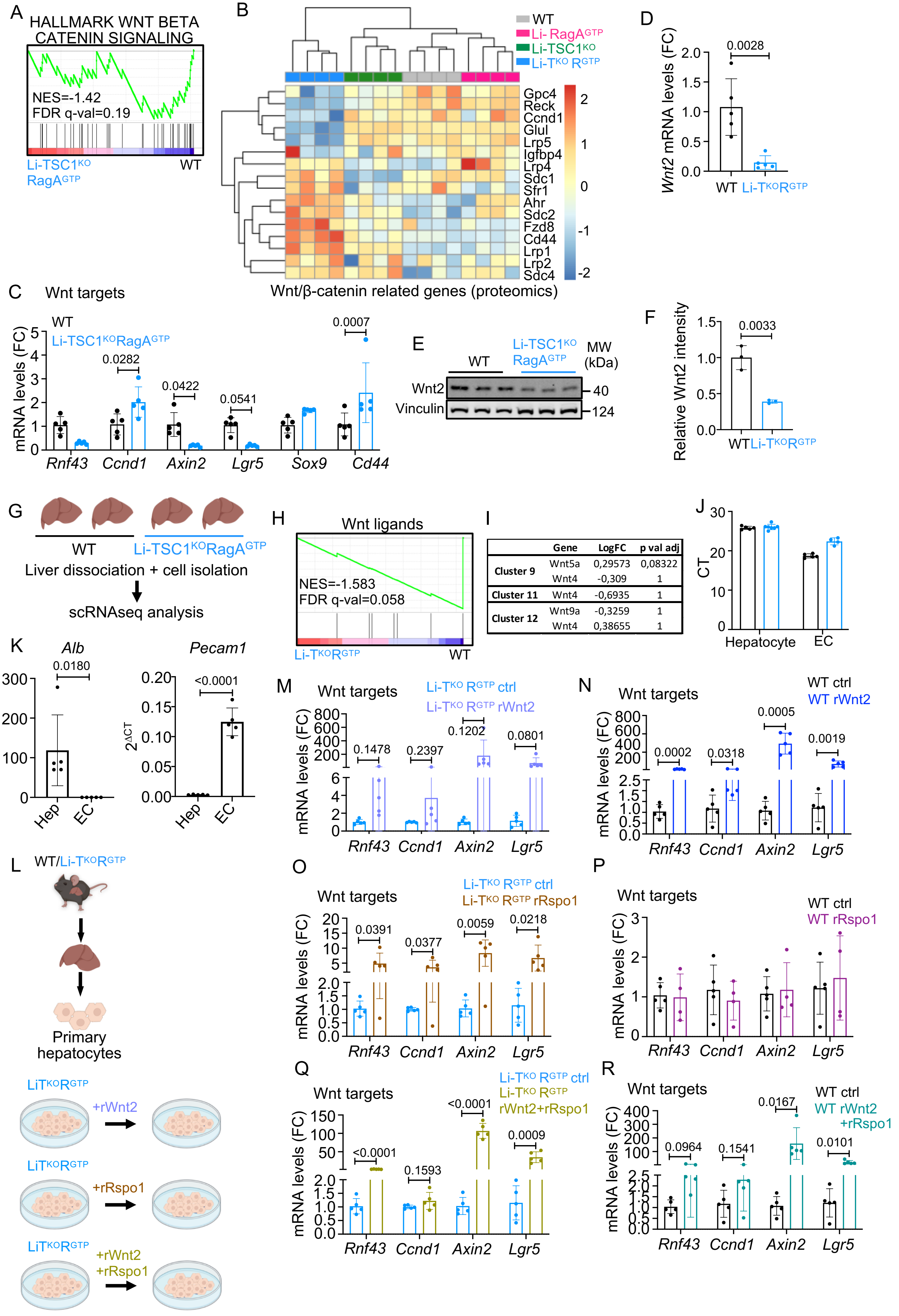
A
HALLMARK WNT BETA CATENIN SIGNALING
NES=-1.42
FDR q-val=0.19
Li-TSC1KO RagAGTP
WT
B
WT
Li- RagAGTP
Li-TSC1KO
Li-TKO RGTP
Gpc4
Reck
Ccnd1
Glul
Lrp5
Igfbp4
Lrp4
Sdc1
Sfr1
Ahr
Sdc2
Fzd8
Cd44
Lrp1
Lrp2
Sdc4
Wnt/β-catenin related genes (proteomics)
D
Wnt2 mRNA levels (FC)
0.0028
WT
Li-TKORGTP
C
Wnt targets
WT
Li-TSC1KORagAGTP
mRNA levels (FC)
0.0282
0.0422
0.0541
0.0007
Rnf43
Ccnd1
Axin2
Lgr5
Sox9
Cd44
E
WT
Li-TSC1KO RagAGTP
MW (kDa)
Wnt2
Vinculin
40
124
F
Relative Wnt2 intensity
0.0033
WT
Li-TKORGTP
G
WT
Li-TSC1KORagAGTP
Liver dissociation + cell isolation
scRNAseq analysis
H
Wnt ligands
NES=-1.583
FDR q-val=0.058
Li-TKORGTP
WT
I
Gene | LogFC | p val adj
Cluster 9 | Wnt5a | 0,29573 | 0,08322
Cluster 9 | Wnt4 | -0,309 | 1
Cluster 11 | Wnt4 | -0,6935 | 1
Cluster 12 | Wnt9a | -0,3259 | 1
Cluster 12 | Wnt4 | 0,38655 | 1
J
CT
Hepatocyte
EC
K
Alb
Pecam1
2ΔCT
0.0180
<0.0001
Hep
EC
L
WT/Li-TKORGTP
Primary hepatocytes
LiTKORGTP
+rWnt2
+rRspo1
+rWnt2 +rRspo1
M
Wnt targets
Li-TKO RGTP ctrl
Li-TKO RGTP rWnt2
mRNA levels (FC)
0.1478
0.2397
0.1202
0.0801
N
Wnt targets
WT ctrl
WT rWnt2
0.0002
0.0318
0.0005
0.0019
O
Wnt targets
Li-TKO RGTP ctrl
Li-TKO RGTP rRspo1
0.0391
0.0377
0.0059
0.0218
P
Wnt targets
WT ctrl
WT rRspo1
Q
Wnt targets
Li-TKO RGTP ctrl
Li-TKO RGTP rWnt2+rRspo1
<0.0001
0.1593
<0.0001
0.0009
R
Wnt targets
WT ctrl
WT rWnt2 +rRspo1
0.0964
0.1541
0.0167
0.0101
Rnf43
Ccnd1
Axin2
Lgr5

Supplementary Figure 5. Related to Figure 5. A. Enrichment plot for the “Wnt beta catenin signaling” gene set from Hallmark for Li-TSC1^{KO}RagA^{GTP} *versus* wild-type livers from mice fasted for 24 hours followed by 2 hours of refeeding at the mRNA level. NES: normalized enrichment score; FDR: false discovery rate. **B.** Hierarchical clustering Heatmap diagram representing protein levels of Wnt/β-catenin related genes from Li-TSC1^{KO}RagA^{GTP} (n=4), Li-TSC1^{KO} (n=4), Li-RagA^{GTP} (n=4) and wild-type (n=4) livers normalized by z-score. **C.** RT-qPCR of livers from 14- to 21-week-old wild-type (n=5) and Li-TSC1^{KO}RagA^{GTP} (n=5) male mice. Expression levels of the indicated Wnt target genes relative to the average level in control mice. β-actin was used as housekeeping gene. Statistical significance was calculated by using multiple unpaired t-test. **D.** RT-qPCR of livers from 14- to 21-week-old wild-type (n=5) and Li-TSC1^{KO}RagA^{GTP} (n=5) male mice. Expression levels of Wnt2 relative to the average level in wild-type mice. β-actin was used as housekeeping gene. Statistical significance was calculated by using unpaired two-tailed t-test. **E.** 19- to 25-week-old wild-type (n=3) and Li-TSC1^{KO}RagA^{GTP} (n=3) female mice were sacrificed. Protein lysates from the liver were immunoblotted for Wnt2. **F.** Levels of Wnt2 on each lane from E are relative to vinculin levels and presented normalized to the average level of wild-type mice. Statistical significance was calculated by using unpaired two-tailed t-test. **G.** Schematic representation of the experimental setting for liver scRNA sequencing from 4-week-old female mice. Number of samples in each condition is indicated for the two genotypes. Picture generated with BioRender. **H.** GSEA related to the signature “Wnt ligands” in transcriptomics from clusters 0+6 (LECs) in livers from wild-type (n=2) and Li-TSC1^{KO}RagA^{GTP} (n=2) mice. NES: normalized enrichment score; FDR: false discovery rate. **I.** Wnt ligands detected in rest of cell types not belonging to EC clusters in scRNAseq analysis from Li-TSC1^{KO}RagA^{GTP} *versus* wild-type livers. **J.** Cycle threshold (CT) of Wnt2 gene in hepatocytes and EC from Li-TSC1^{KO}RagA^{GTP} *versus* wild-type livers. **K.** Validation of the purity of the hepatocyte and endothelial cell purification. Albumin and Pecam1 were used as markers of hepatocytes (Hep) and endothelial cells (EC), respectively. **L.** Primary hepatocytes isolated from Li-TSC1^{KO}RagA^{GTP} mice were supplemented with mouse rWnt2, mouse rRspo1 or both. Picture generated with BioRender. **M.** RT-qPCR of primary hepatocytes from 7-week-old Li-TSC1^{KO}RagA^{GTP} mice (n=5) supplemented with rWnt2 for 16 hours. Expression levels of the indicated Wnt target genes relative to the average level in control mice. β-actin was used as housekeeping gene. Statistical significance was calculated by using 2way ANOVA with Sidák’s multiple comparisons test. **N.** RT-qPCR of primary hepatocytes from 7- to 9-week-old wild-type mice (n=5) supplemented with rWnt2 for 16 hours. Expression levels of the indicated Wnt target genes relative to the average level in control mice. β-actin was used as housekeeping gene. Statistical significance was calculated by using 2way ANOVA with Sidák’s multiple comparisons test. **O.** RT-qPCR of primary hepatocytes from 7-week-old Li-TSC1^{KO}RagA^{GTP} mice (n=5) supplemented with rRspo1 for 16 hours. Expression levels of the indicated Wnt target genes relative to the average level in control mice. β-actin was used as housekeeping gene. Statistical significance was calculated by using 2way ANOVA with Sidák’s multiple comparisons test. **P.** RT-qPCR of primary hepatocytes from 7- to 9-week-old wild-type mice (n=5) supplemented with rRspo1 for 16 hours. Expression levels of the indicated Wnt target genes relative to the average level in control mice. β-actin was used as housekeeping gene. Statistical significance was calculated by using 2way ANOVA with Sidák’s multiple comparisons test. **Q.** RT-qPCR of primary hepatocytes from 7-week-old Li-TSC1^{KO}RagA^{GTP} mice (n=5) supplemented with rWnt2 and rRspo1 for 16 hours. Expression levels of the indicated Wnt target genes relative to the average level in control mice. β-actin was used as housekeeping gene. Statistical significance was calculated by using 2way ANOVA with Sidák’s multiple comparisons test. **R.** RT-qPCR of primary hepatocytes from 7- to 9-week-old wild-type mice (n=5) supplemented with rWnt2 and rRspo1 for 16 hours. Expression levels of the indicated Wnt target genes relative to the average level in control mice. β-actin was used as housekeeping gene. Statistical significance was calculated by using 2way ANOVA with Sidák’s multiple comparisons test.

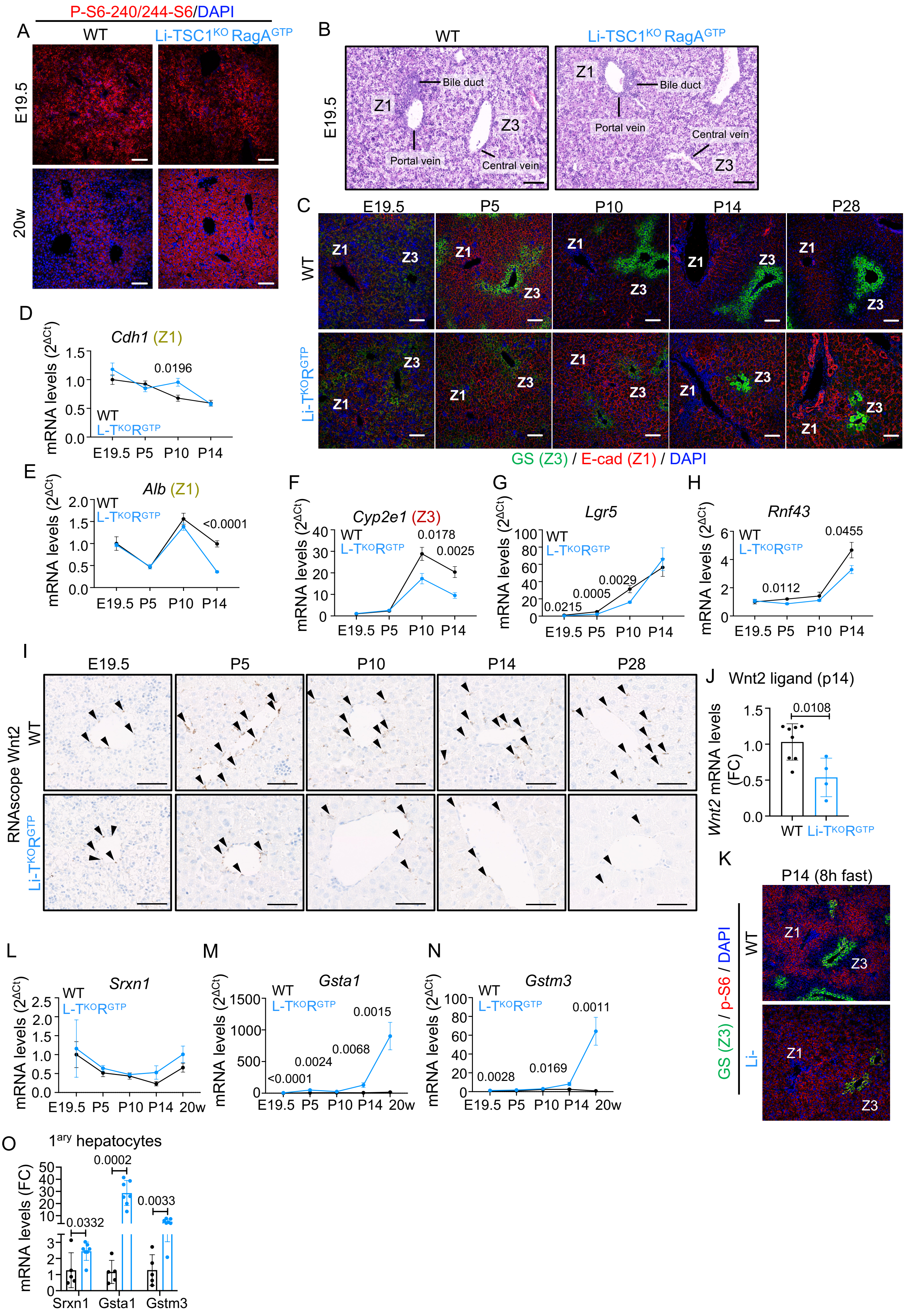

P-S6-240/244-S6/DAPI
A
WT
Li-TSC1KO RagAGTP
E19.5
20w
B
WT
Li-TSC1KO RagAGTP
E19.5
Bile duct
Z1
Z3
Portal vein
Central vein
C
E19.5
P5
P10
P14
P28
WT
Li-TKORGTP
GS (Z3) / E-cad (Z1) / DAPI
D
Cdh1 (Z1)
0.0196
mRNA levels (2ΔCt)
WT
L-TKORGTP
E19.5 P5 P10 P14
E
Alb (Z1)
<0.0001
F
Cyp2e1 (Z3)
0.0178
0.0025
G
Lgr5
0.0215
0.0005
0.0029
H
Rnf43
0.0112
0.0455
I
RNAscope Wnt2
E19.5
P5
P10
P14
P28
J
Wnt2 ligand (p14)
0.0108
Wnt2 mRNA levels (FC)
WT Li-TKORGTP
K
P14 (8h fast)
GS (Z3) / p-S6 / DAPI
WT
Li-
L
Srxn1
E19.5 P5 P10 P14 20w
M
Gsta1
<0.0001
0.0024
0.0068
0.0015
N
Gstm3
0.0028
0.0169
0.0011
O
1ary hepatocytes
mRNA levels (FC)
0.0332
0.0002
0.0033
Srxn1 Gsta1 Gstm3

Supplementary Figure 6. Related to Figure 6. A. Representative pictures of immunofluorescence against Phospho-S240-244-S6 in the liver of wild-type and Li-TSC1^{KO}RagA^{GTP} mice at E19.5 and in adult mice fasted during 24 hours. Scale bar 100 µm. **B.** Representative hepatic H&E staining of wild-type and Li-TSC1^{KO}RagA^{GTP} mice at E19.5. Scale bar 100 µm. **C.** Representative pictures of immunofluorescence against Glutamine synthetase (Zone 3) together with E-cadherin (Zone 1) in the liver of wild-type and Li-TSC1^{KO} RagA^{GTP} mice at E19.5, P5, P10, P14 and P28. Zone 1 and Zone 3 are highlighted in each image. Scale bar 100 µm. **D-H.** RT-qPCR of livers from E19.5 wild-type (n=6) and Li-TSC1^{KO}RagA^{GTP} (n=5) mice, p5 wild-type (n=7) and Li-TSC1^{KO}RagA^{GTP}(n=7) mice, p10 wild-type (n=4) and Li-TSC1^{KO}RagA^{GTP}(n=6) mice, and p14 wild-type (n=7) and Li-TSC1^{KO}RagA^{GTP}(n=7) mice relative to levels in E19.5 wild-type animals. β-actin was used as housekeeping gene. Data are shown as mean with SEM. Statistical significance was calculated by using multiple unpaired t-test. **I.** Representative pictures of RNAscope against mouse Wnt2 in the liver of E19.5, p5, p10, p14 and p28 wild-type and Li-TSC1^{KO}RagA^{GTP} mice. Scale bar 20 µm. Arrows point EC with mWnt2 positive staining. **J.** RT-qPCR of EC at time 0 from p14 wild-type (n=8) and Li-TSC1^{KO}RagA^{GTP} (n=4) mice. Expression levels of Wnt2 relative to the average level in wild-type mice. β-actin was used as housekeeping gene. Statistical significance was calculated by using unpaired two-tailed t-test. **K.** Representative pictures of immunofluorescence against Glutamine Synthetase together with Phospho-S240/244-S6 in the liver of p14 wild-type and Li-TSC1^{KO} RagA^{GTP} mice fasted for 8 hours. Zone 1 and Zone 3 are highlighted in each image. Scale bar 100 µm. **L-N.** RT-qPCR of livers from E19.5 wild-type (n=6) and Li-TSC1^{KO}RagA^{GTP} (n=5) mice, p5 wild-type (n=7) and Li-TSC1^{KO}RagA^{GTP}(n=7) mice, p10 wild-type (n=4) and Li-TSC1^{KO}RagA^{GTP}(n=6) mice, and p14 wild-type (n=7) and Li-TSC1^{KO}RagA^{GTP}(n=7) mice relative to levels in E19.5 wild-type animals. β-actin was used as housekeeping gene. Data are shown as mean with SEM. Statistical significance was calculated by using multiple unpaired t-test. **O.** RT-qPCR of primary hepatocytes at time 0 from 7-week-old wild-type (n=5) and Li-TSC1^{KO}RagA^{GTP} (n=7) mice. Expression levels of the indicated genes relative to the average level in wild-type mice. β-actin was used as housekeeping gene. Statistical significance was calculated by using multiple unpaired t-test.

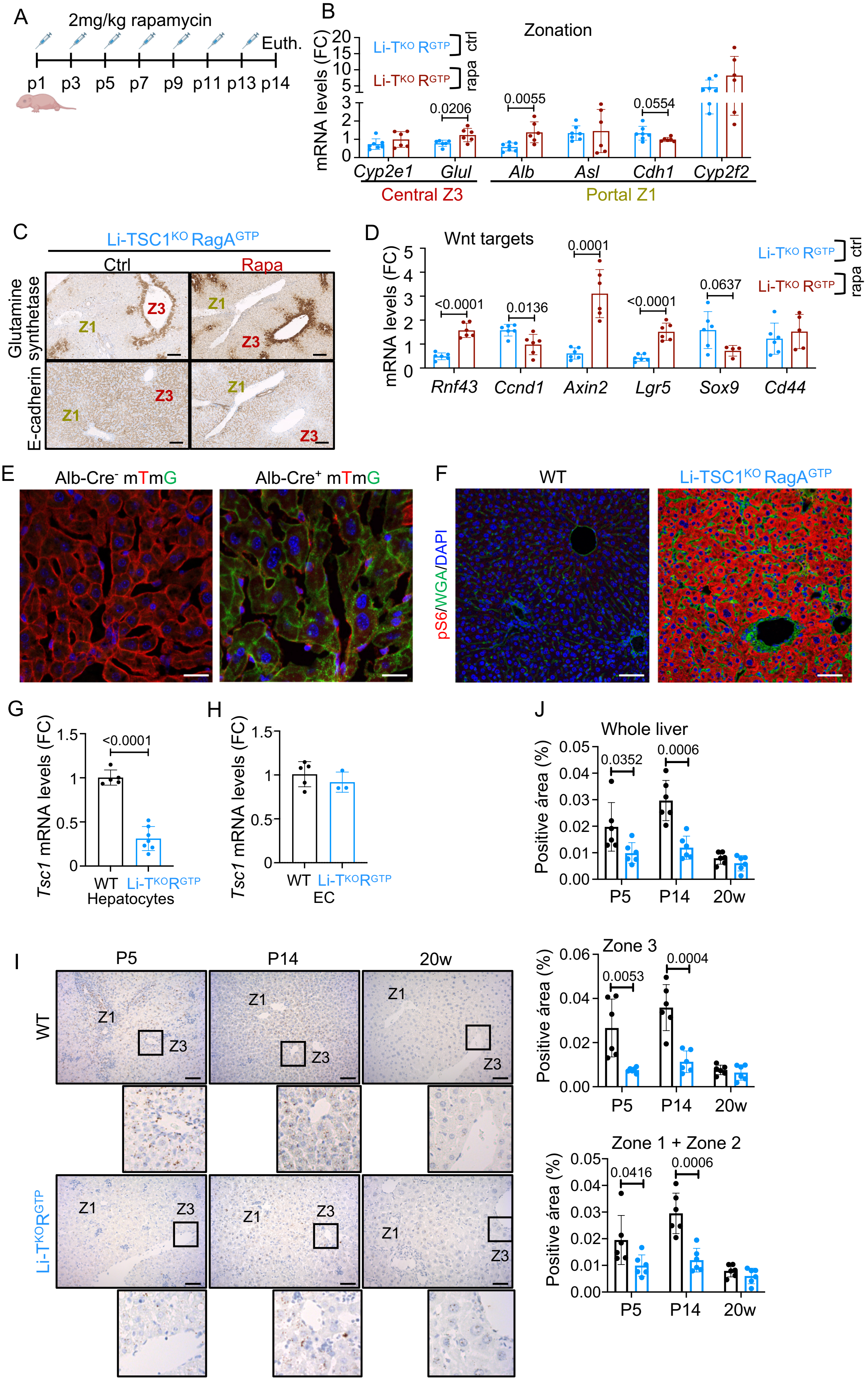

A
2mg/kg rapamycin
Euth.
p1 p3 p5 p7 p9 p11 p13 p14
B
Zonation
mRNA levels (FC)
Li-TKO RGTP ctrl
Li-TKO RGTP rapa
0.0206
0.0055
0.0554
Cyp2e1 Glul Alb Asl Cdh1 Cyp2f2
Central Z3
Portal Z1
C
Li-TSC1KO RagAGTP
Ctrl
Rapa
Glutamine synthetase
E-cadherin
Z1
Z3
D
Wnt targets
mRNA levels (FC)
<0.0001
0.0136
0.0001
<0.0001
0.0637
Rnf43 Ccnd1 Axin2 Lgr5 Sox9 Cd44
E
Alb-Cre- mTmG
Alb-Cre+ mTmG
F
WT
Li-TSC1KO RagAGTP
pS6/WGA/DAPI
G
Tsc1 mRNA levels (FC)
<0.0001
WT Li-TKORGTP
Hepatocytes
H
Tsc1 mRNA levels (FC)
WT Li-TKORGTP
EC
J
Whole liver
Positive área (%)
0.0352
0.0006
P5 P14 20w
Zone 3
0.0053
0.0004
Zone 1 + Zone 2
0.0416
0.0006
I
P5 P14 20w
WT
Li-TKORGTP

Supplementary Figure 7. Related to Figure 6. A. Rapamycin was administered to Li-TSC1KO RagAGTP mice at p1 and through a 2-week period, when the mice were euthanized. Picture generated with BioRender. **B.** RT-qPCR of livers from p14 Li-TSC1^{KO}RagA^{GTP} control (n=7) and Li-TSC1^{KO}RagA^{GTP} rapamycin (n=6) mice. Expression levels of the indicated genes involved in zonation relative to the average level in wild-type control mice. β-actin was used as housekeeping gene. Statistical significance was calculated by using 2way ANOVA with Tukey's multiple comparisons test. **C.** Representative pictures of immunohistochemistry against Glutamine synthetase or E-cadherin in the liver of control and rapamycin-treated p14 Li-TSC1^{KO}RagA^{GTP} mice. Scale bar 100 µm. **D.** RT-qPCR of livers from p14 Li-TSC1^{KO}RagA^{GTP} control (n=7) and Li-TSC1^{KO}RagA^{GTP} rapamycin (n=6) mice. Expression levels of the indicated genes involved in Wnt/β-catenin signaling pathway relative to the average level in wild-type mice in the control condition. β-actin was used as housekeeping gene. Statistical significance was calculated by using 2way ANOVA with Tukey's multiple comparisons test. **E.** Representative pictures of fluorescence of Alb-Cre^{0} mTmG and Alb-Cre^{+} mTmG mice. **F.** Representative pictures of immunofluorescence against membrane marker WGA and Phospho-S235/236-S6 in wild-type and Li-TSC1^{KO}RagA^{GTP} mice fasted for 24 h. **G.** RT-qPCR of primary hepatocytes at time 0 from 7-week-old wild-type (n=5) and Li-TSC1^{KO}RagA^{GTP} (n=7) mice. Ratio between expression levels of loxP-flanked exon 17 and exon 4 of *Tsc1* gene relative to the average level in wild-type mice. β-actin was used as housekeeping gene. Statistical significance was calculated by using unpaired two-tailed t-test. **H.** RT-qPCR of primary LECs at time 0 from 8- to 15-week-old wild-type (n=5) and Li-TSC1^{KO}RagA^{GTP} (n=3) mice. Ratio between expression levels of loxP-flanked exon 17 and exon 4 of *Tsc1* gene relative to the average level in wild-type mice. β-actin was used as housekeeping gene. Statistical significance was calculated by using unpaired two-tailed t-test. **I.** Representative pictures of RNAscope against mouse Fzd8 in the liver of p5, p14 and 20 week-old wild-type and Li-TSC1^{KO}RagA^{GTP} mice. Scale bar 10 µm. **J.** Quantification of positive area of RNAscope against mouse Fzd8 shown in X in whole liver (top), exclusively in Zone 3 (middle) and in Zone 1+2 (down) (6 images from 2 mice per condition).

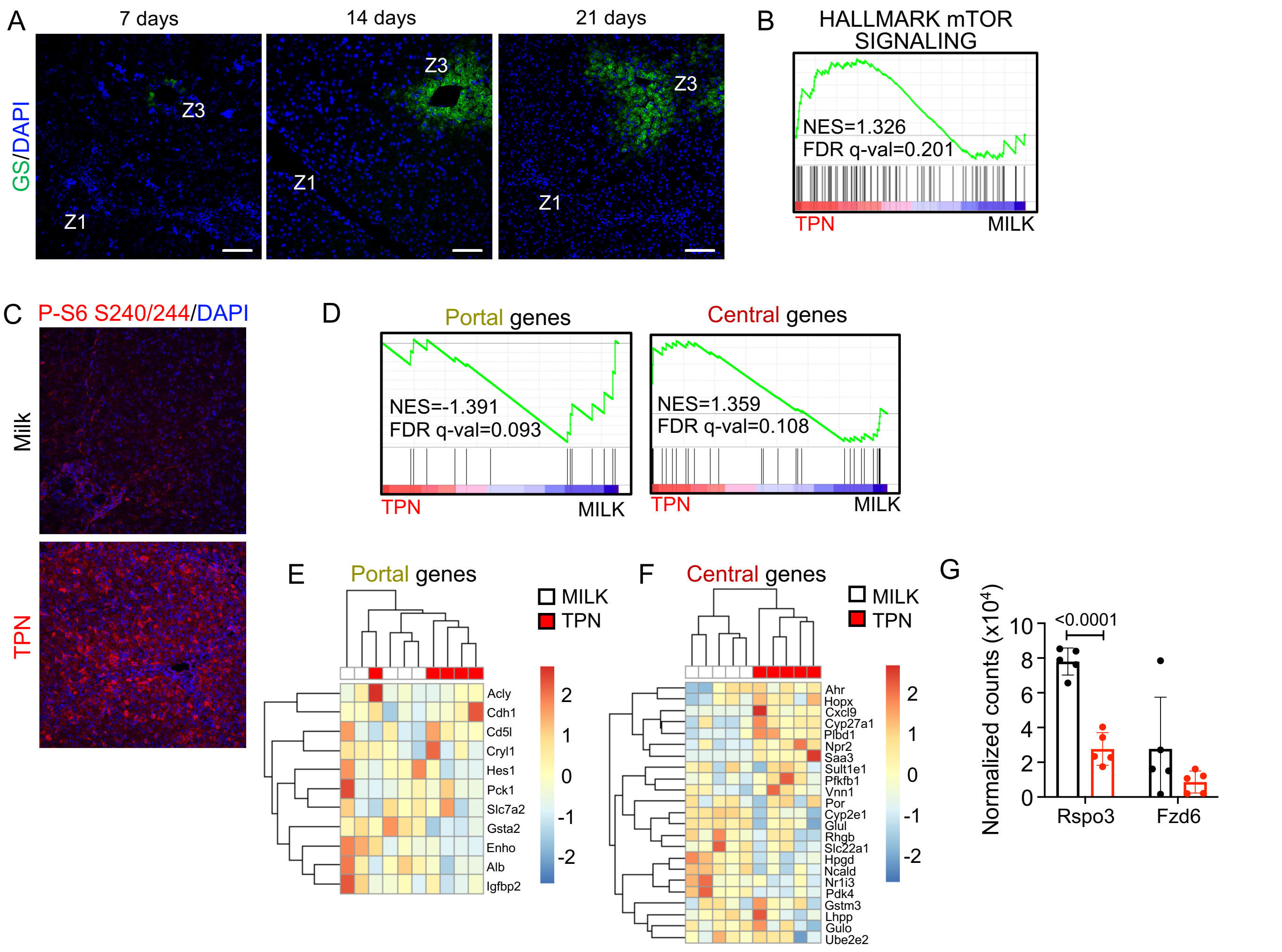

Supplementary Figure 8. Related to Figure 7. A. Representative pictures of immunofluorescence against Glutamine Synthetase in the liver of piglets at 7 days, 14 days and 21 days. Scale bar 100 µm. **B.** Enrichment plot for the "mTOR signaling" gene set from Hallmark for TPN-fed *versus* control livers from piglets at the mRNA level. NES: normalized enrichment score; FDR: false discovery rate. **C.** Representative pictures of immunofluorescence against Phospho-S240-244-S6 in the liver of control and TPN-fed piglets. Scale bar 100 µm. **D.** Enrichment of gene sets related to central and portal signatures in transcriptomics from TPN-fed (n=5) and orally-fed (n=5) piglets. NES: normalized enrichment score; FDR: false discovery rate. **E, F** Hierarchical clustering Heatmap diagrams representing mRNA expression patterns of portal and central genes in livers from TPN-fed (n=5) and orally-fed (n=5) piglets normalized by z-score. **G.** Normalized counts of Rspo3 and Fzd6 genes detected by bulk liver RNA sequencing in MILK (n=5) *versus* TPN (n=5) samples.

Periportal genes (Gene ID)		Pericentral genes (Gene ID)		
Alb	Mafb	Glul	Slc22a3	Cyp3a59
Asl	Tsc22d4	Cyp2e1	Slc13a3	Cyp2c54
Ass1	Slc7a2	Gck	Slc1a2	Hip1r
Cdh1	Cyp2u1	Cyp7a1	Cib2	Rnf43
Cyp2f2	Scnn1a	Cyp27a1	Npr2	Gbp11
Sds	Aspg	Lgr5	Cyp2c37	Sult1e1
Pck1	Slc13a2	Oat	Lhpp	Fam89a
Sult5a1	Gls2	Pfkfb1	Cyp2a5	Fam82a1
Aldh1b1	Mfsd2a	Cyp1a2	Slc1a4	Hpgd
Acly	Celsr1	Cyp7a1	Fam55b	Acot1
Ctsc	Bdh2	Gpr49	Pcp4l1	Cyp2a22
Hes1	Mup20	Nr1i3	Sntb1	Vnn1
Ctnnbip1	Cyp17a1	Ahr	Lect2	Pdk4
Gsta2	Gpc1	Rhbg	Axin2	Ncald
As3mt	Gas2	Cyp2a4	Tbx3	Akr1c20
Cux2	Agxt2l1	Cyp2a5	Plbd1	Cyp2d40
Cyp4a12	Sdsl	Gstm3	Ces2c	Saa3
Adrbk2	Ugt2b38	Gstm2	Gulo	Slc16a7
Tbc1d30	Hsd17b6	Ces2	Cxcl9	Akr1b7
Clec4f	Cryl1	Gstm6	Endod1	Gda
Serpina4-ps1	Serpina12	Cyp2c50	Adh6-ps1	Lcn2
Enho	Mmd2	Cyp2c55	Rec8	Hopx
Cobl	Clec2h	Cyp2c29	Slc22a1	Orm2
Cd5l	Setd7	Cyp2g1	Ndrg1	Cxcl1
Igfbp2		Por	Sult1b1	Scd2
		Cyp2c38	Cyp2c39	Slc1a5
		Gsta3	Slc16a10	Saa2
		Sult1b1	Ube2e2	Ugt2b37
		Gstm1	Blvrb	

Supplementary Table 1. List of curated genes related to liver zonation.

Ahr	Glul	Lrp2bp	Sdc2
Ascl2	Gpc1	Lrp3	Sdc3
Axin2	Gpc3	Lrp4	Sdc4
Ccnd1	Gpc4	Lrp5	Sfr1
Cd44	Igfbp4	Lrp6	Sfrp1
Dkk3	Kremen1	Myc	Sfrp5
Fzd1	Lgr4	Notum	Sox9
Fzd3	Lgr5	Reck	Wnt2
Fzd4	Lrp1	Rnf43	Wnt4
Fzd5	Lrp10	Ror1	Wnt5a
Fzd6	Lrp11	Rspo1	Wnt5b
Fzd7	Lrp12	Rspo3	Wnt9b
Fzd8	Lrp1b	Ryk	Znrf3
Fzd9	Lrp2	Sdc1	

Supplementary Table 2. List of curated genes related to Wnt pathway.
