# Supplementary material for: Hepatic nutrient and hormone signaling to mTORC1 instructs the postnatal metabolic zonation of the liver
Source: Nat Commun. 2024 Mar 18;15:1878. doi: 10.1038/s41467-024-46032-1 (PMC10948770; doi:10.1038/s41467-024-46032-1)
Supplement: Supplementary file 3 — Description of Additional Supplementary Files [file 41467_2024_46032_MOESM3_ESM.pdf]

Description of Additional Supplementary Files

File Name: Supplementary Data 1
Description: Raw data of RNAseq, scRNAseq and proteomics data from this study.

File Name: Supplementary Data 2
Description: Primer sequences used in this study.
