# Supplementary material for: Hepatic nutrient and hormone signaling to mTORC1 instructs the postnatal metabolic zonation of the liver
Source: Nat Commun. 2024 Mar 18;15:1878. doi: 10.1038/s41467-024-46032-1 (PMC10948770; doi:10.1038/s41467-024-46032-1)
Supplement: Supplementary file 6 — Reporting Summary [file 41467_2024_46032_MOESM6_ESM.pdf]

Corresponding author(s): Alejo Efeyan

Last updated by author(s): 24/01/24

Reporting Summary

Nature Portfolio wishes to improve the reproducibility of the work that we publish. This form provides structure for consistency and transparency in reporting. For further information on Nature Portfolio policies, see our Editorial Policies and the Editorial Policy Checklist.

Statistics

For all statistical analyses, confirm that the following items are present in the figure legend, table legend, main text, or Methods section.

n/a	Confirmed	
☐	☒	The exact sample size (*n*) for each experimental group/condition, given as a discrete number and unit of measurement
☐	☒	A statement on whether measurements were taken from distinct samples or whether the same sample was measured repeatedly
☐	☒	The statistical test(s) used AND whether they are one- or two-sided<br>*Only common tests should be described solely by name; describe more complex techniques in the Methods section.*
☒	☐	A description of all covariates tested
☐	☒	A description of any assumptions or corrections, such as tests of normality and adjustment for multiple comparisons
☐	☒	A full description of the statistical parameters including central tendency (e.g. means) or other basic estimates (e.g. regression coefficient) AND variation (e.g. standard deviation) or associated estimates of uncertainty (e.g. confidence intervals)
☐	☒	For null hypothesis testing, the test statistic (e.g. *F*, *t*, *r*) with confidence intervals, effect sizes, degrees of freedom and *P* value noted<br>*Give P values as exact values whenever suitable.*
☒	☐	For Bayesian analysis, information on the choice of priors and Markov chain Monte Carlo settings
☒	☐	For hierarchical and complex designs, identification of the appropriate level for tests and full reporting of outcomes
☒	☐	Estimates of effect sizes (e.g. Cohen's *d*, Pearson's *r*), indicating how they were calculated

Our web collection on statistics for biologists contains articles on many of the points above.

Software and code

Policy information about availability of computer code

Data collection	Odyssey Infrared Imaging System (Application software version 3.0.30) LI-COR Biosciences - v10, AxioScan Z1, Zeiss - Zen 2.3 Blue edition Software (Zeiss)
Data analysis	GraphPad v9

For manuscripts utilizing custom algorithms or software that are central to the research but not yet described in published literature, software must be made available to editors and reviewers. We strongly encourage code deposition in a community repository (e.g. GitHub). See the Nature Portfolio guidelines for submitting code & software for further information.

Data

Policy information about availability of data

All manuscripts must include a data availability statement. This statement should provide the following information, where applicable:

- Accession codes, unique identifiers, or web links for publicly available datasets
- A description of any restrictions on data availability
- For clinical datasets or third party data, please ensure that the statement adheres to our policy

The transcriptomics data generated in this study have been deposited to the GEO database under the following accession codes: bulk liver from Li-TSC1KORagAGTPmice (GSE225265), bulk liver from control and TPN-fed neonatal pigs (GSE225266), scRNAseq from Li-TSC1KORagAGTP and wild-type livers (GSE229830) and endothelial cells isolated from wild-type and Li-TSC1KORagAGTP livers (GSE241848). The mass spectrometry proteomics data have been deposited

to the ProteomeXchange Consortium with the identifier PXD041439. The authors declare that other source data supporting the findings of this study within the article and its Supplementary information files are available upon reasonable request to the corresponding author. Source data are provided with this paper.

Research involving human participants, their data, or biological material

Policy information about studies with human participants or human data. See also policy information about sex, gender (identity/presentation), and sexual orientation and race, ethnicity and racism.

Reporting on sex and gender	not applicable - research without human particpants, their data or biological material
Reporting on race, ethnicity, or other socially relevant groupings	not applicable - research without human particpants, their data or biological material
Population characteristics	not applicable - research without human particpants, their data or biological material
Recruitment	not applicable - research without human particpants, their data or biological material
Ethics oversight	not applicable - research without human particpants, their data or biological material

Note that full information on the approval of the study protocol must also be provided in the manuscript.

Field-specific reporting

Please select the one below that is the best fit for your research. If you are not sure, read the appropriate sections before making your selection.

☒ Life sciences ☐ Behavioural & social sciences ☐ Ecological, evolutionary & environmental sciences

For a reference copy of the document with all sections, see nature.com/documents/nr-reporting-summary-flat.pdf

Life sciences study design

All studies must disclose on these points even when the disclosure is negative.

Sample size	No sample size calculation was performed, as the magnitude of the effect sizes were unknown. As reference, the sample sizes were guided on the basis of similar published studies (PMID: 24768164, PMID 21179166, PMID: 34135321).<br>Details on sample size of all experiments are provided in the Methods section and figure legends.
Data exclusions	No data were excluded unless as determined by technical problems.
Replication	All attempts of replication (at least twice) under independent conditions were successful.
Randomization	Mice, cells and samples were randomly assigned to different treatments/conditions.
Blinding	Experiments were not blinded as double mutant mice were sometimes distinguishable by eye. For experiments other than those involving mice, investigators were blinded to group allocation during data collection and/or analysis.

Reporting for specific materials, systems and methods

We require information from authors about some types of materials, experimental systems and methods used in many studies. Here, indicate whether each material, system or method listed is relevant to your study. If you are not sure if a list item applies to your research, read the appropriate section before selecting a response.

Materials & experimental systems

n/a	Involved in the study	
☐	☒	Antibodies
☒	☐	Eukaryotic cell lines
☒	☐	Palaeontology and archaeology
☐	☒	Animals and other organisms
☒	☐	Clinical data
☒	☐	Dual use research of concern
☒	☐	Plants

Methods

n/a	Involved in the study	
☒	☐	ChIP-seq
☒	☐	Flow cytometry
☒	☐	MRI-based neuroimaging

Antibodies

Antibodies used

P-T389-S6K1 (CST #9234)
S6K1 (CST #2708)
P-S235/236-S6 (CST #2211)
S6 (CST #2217)
P-T37/46-4EBP1 (CST #2855)
4EBP1 (CST #9644)
RagA (CST #4357)
TSC1 (CST #6935)
Vinculin (Sigma #V9131)
CD45 (CST #70257)
Glutamine synthetase (SIGMA #G2781)
E-cadherin (BD Biosciences #610182)
Phospho-S240/244-S6 (CST #5364)
Wheat Germ Agglutinin, Alexa Fluor 488 conjugate (Thermo Fisher #W11261)
CD144 antibody (BD Biosciences #555289).
Ornithine aminotransferase (GeneTex #GTX50004)
Phosphoenolpyruvate Carboxykinase 1 (Ptglab #16754-1-AP)
Alexa fluor 555 goat anti-rabbit (Life Technologies #A21428)
Alexa fluor chicken anti-mouse (Life Technologies #A21200)

Validation

P-T389-S6K1 (#9234) CST - Used for WB in primary hepatocytes as validated by the company and by users (cited 1754 times)
S6K1 (#2708) CST - Used for WB in primary hepatocytes as validated by the company and by users (cited 1551 times)
P-S235/236-S6 (#2211) CST - Used for WB in mouse tissues as validated by the company and by users (cited 1446 times)
S6 (#2217) CST - Used for WB in mouse tissues as validated by the company and by users(cited 2158 times)
P-T37/46-4EBP1 (#2855) CST - Used for WB in mouse tissues as validated by the company and by users (cited 1684 times)
4EBP1 (#9644) CST - Used for WB in mouse tissues as validated by the company and by users (cited 1166 times)
RagA (#4357) CST - Used for WB in mouse tissues as validated by the company and by users (cited 79 times)
TSC1 (#6935) CST – Used for WB in mouse tissues as validated by the company and by users (cited 94 times)
Vinculin (#V9131) Sigma - Used for WB in mouse tissues as validated by the company and by users (cited 1744 times)
CD45 (#70257) – CST – Used for immunohistochemistry in mouse tissues as validated by the company and by users (cited 43 times)
Glutamine synthetase (SIGMA #G2781) – Used for immunohistochemistry in mouse tissues as validated by the company and by users (cited 85 times)
E-cadherin (BD Biosciences #610182) - Used for immunohistochemistry and immunofluorescence in mouse tissues as validated by the company and by users (cited 890 times)
Phospho-S240/244-S6 (CST #5364) - Used for immunofluorescence in mouse tissues as validated by the company and by users (cited 786 times)
Wheat Germ Agglutinin, Alexa Fluor 488 conjugate (Thermo Fisher #W11261) - Used for immunofluorescence in mouse tissues as validated by the company and by users (cited 35 times)
CD144 antibody (BD Biosciences #555289) - Used for isolation of primary endothelial cells and as validated by the company and by users (cited 148 times)
Ornithine aminotransferase (GeneTex #GTX50004) - Used for western blot, ICC/IF, FACS, ELISA as validated by the company. Currently no references.
Phosphoenolpyruvate Carboxykinase 1 (Ptglab #16754-1-AP) - used for western blot, immunoprecipitation, immunofluorescence as validated by the company and by users (cited 59 times)
Alexa fluor 555 goat anti-rabbit (Life Technologies #A21428) - used for immunohistochemistry, immunocytochemistry (ICC/IF) and flow citometry as validated by the company and by users (cited 1459 times)
Alexa fluor chicken anti-mouse (Life Technologies #A21200) - used for western blot, immunohistochemistry, immunocitochemistry (ICC/IF) as validated by the company and by users (cited 282 times)

Animals and other research organisms

Policy information about studies involving animals; ARRIVE guidelines recommended for reporting animal research, and Sex and Gender in Research

Laboratory animals

Mus musculus. For hepatocyte-specific activation of RagA (Li-RagAGTP), RragAGTP/flox mice 17 were bred with mice carrying Albumin-Cre (Alb-Cre) recombinase 56 (JAX stock #003574). For hepatocyte-specific deletion of Tsc1 (Li-TSC1KO), Tsc1flox/flox mice 57 (JAX stock #005680) were bred with mice carrying Albumin-Cre (Alb-Cre) recombinase 56 (JAX stock #003574). For the generation of mice with constitutive nutrient and growth factor signaling to mTORC1 (Li-TSC1KORagAGTP) we crossed Albumin-Cre RragAGTP/flox mice with Tsc1flox/flox mice. For the validation of absence of Alb-Cre mediated recombination in liver endothelial cells, we bred Albumin-Cre mice with mice carrying the Gt(ROSA)26Sortm4(ACTB-tdTomato,-EGFP)Luo (mTmG) allele 58 (JAX stock #007676). Male and female mice from E19.5 to 2.9 years were used. Mice were housed under specific pathogen free conditions at 22°C and with 12-h dark/light cycles (light cycle from 8:00 to 20:00). Mice were fed with a standard chow diet (Harlan Teklad #2018S/2018SC). Sex was considered in the study design and analysis of metabolic experiments. All animal procedures carried out at the CNIO were performed according to protocols approved by the CNIO-ISCIII Ethics Committee for Research and Animal Welfare (CEIyBA) and the Autonomous Community of Madrid (CAM). Protocol numbers PROEX285/15, PROEX15/18 and PROEX225.7/22.

Sus-scrofa/domestica. 2-week-old male piglets (strain White Yorkshire x Landrace pigs) were used in this study.

Wild animals

not used

Reporting on sex	Experiments where performed on both males and females unless indicated
Field-collected samples	No field-collected samples
Ethics oversight	All animal procedures carried out at the CNIO were performed according to protocols approved by the CNIO-ISCIII Ethics Committee for Research and Animal Welfare (CEIyBA) and the Autonomous Community of Madrid (CAM). Protocol numbers PROEX285/15, PROEX15/18 and PROEX225.7/22.

Note that full information on the approval of the study protocol must also be provided in the manuscript.
